# Association Between Carotid Artery Small Plaque on Computed Tomography Angiography and Embolic Stroke of Undetermined Source

**DOI:** 10.3390/neurolint17090148

**Published:** 2025-09-14

**Authors:** Junpei Nagasawa, Tatsuhiro Yokoyama, Makiko Ogawa, Ryuichi Okamoto, Mari Shibukawa, Junya Ebina, Takehisa Hirayama, Osamu Kano

**Affiliations:** Department of Neurology, Toho University Faculty of Medicine, Tokyo 143-8541, Japan; n.junpei62@gmail.com (J.N.);

**Keywords:** carotid artery small plaque, embolic stroke of undetermined source, computed tomography angiography, unstable plaque

## Abstract

**Objectives**: While traditionally, carotid plaques with significant stenosis have been considered major embolic sources, recent evidence suggests that even non-stenotic small plaques with a <50% stenosis rate may contribute to cerebral infarction. Herein, we evaluated the relationship between non-stenotic small plaques and embolic stroke of undetermined source (ESUS) using computed tomography angiography (CTA). **Materials and Methods**: We retrospectively reviewed our single-institutional database of hospitalized patients with stroke between April 2017 and December 2022 and enrolled them with ESUS. We evaluated the presence or absence of non-stenotic carotid artery plaque lesions ipsilateral and contralateral to the cerebral infarction lesion using CTA. A neurologist, blinded to the stroke side and all other clinical information, reviewed each CTA and viewed the axial and sagittal CTA source images. In each image, a line perpendicular to the vessel wall was drawn and the plaque diameter was measured. The largest part was considered as the maximum plaque diameter. **Results**: A total of 951 patients with stroke were hospitalized during the study period. Among these, 35 patients with unilateral anterior circulation ESUS were enrolled. Plaque prevalence > 3 mm was compared between the carotid artery on the ESUS side and contralateral carotid artery. The prevalences were 31% and 8% on the ESUS and contralateral sides, respectively. Plaques > 3 mm were often found on the ESUS side. **Conclusions**: Patients with ESUS were more likely to exhibit non-stenotic plaques of ≥3 mm in the infarcted carotid artery than in the contralateral carotid artery. Thus, small non-stenotic plaques may be the embolization source in ESUS, and CT angiography is useful for these evaluations.

## 1. Introduction

Embolic stroke of undetermined source (ESUS) represents nearly one-sixth of ischemic strokes and is associated with a higher likelihood of recurrence compared with other subtypes [1].

Therefore, it is necessary to consider further effective preventive strategies that are tailored to the specific underlying embolic source. In earlier hypotheses, the majority of embolic sources in ESUS were believed to be occult paroxysmal atrial fibrillation (Paf) [2]. Based on this assumption, it was proposed that anticoagulant therapy might surpass antiplatelet agents in preventing stroke recurrence among individuals with ESUS [3].

Nevertheless, both the NAVIGATE ESUS and RE-SPECT ESUS trials, which evaluated direct oral anticoagulants against aspirin in ESUS patients, failed to show a superiority of direct-acting oral anticoagulants (DOACs) over aspirin [4,5].

These findings suggest that subclinical atrial fibrillation may not be the predominant cause of ESUS and that alternative mechanisms such as arteriogenic cerebral embolism may play a more significant role. Moreover, even with prolonged cardiac rhythm monitoring using an insertable cardiac monitor (ICM), Paf is detected in only approximately 23% of patients with ESUS [6], indicating that other embolic sources may be more prevalent. Atrial cardiopathy, involving structural or functional abnormalities of the left atrium, has been suggested as a cause of ESUS without detected atrial fibrillation [7]. Markers like PTFV1, left atrial size, and NT-proBNP help identify it. The ARCADIA trial tested anticoagulants but found no clear benefit over aspirin, so its clinical importance remains uncertain [8].

Other potential causes of ESUS include low-risk cardioembolic sources, paradoxical embolism through a patent foramen ovale (PFO), cancer-associated stroke, and aortic arch atheroma. However, an increasing number of reports suggest that arteriogenic cerebral embolism may be more prevalent than previously thought [9].

Stenosis of ≥50% is required to determine whether cerebral infarction is caused by carotid atherosclerotic disease according to the commonly used classification criteria for cerebral infarction [10]. Even in the diagnostic criteria for ESUS, stenosis of <50% in proximal vessels is not considered an embolism source [11].

However, even non-stenotic small plaques with a stenosis rate of <50% have been recently reported to become embolic sources of cerebral infarction [12]. This concept has been formalized as symptomatic non-stenotic carotid disease (SyNC), in which non-stenotic plaques cause symptomatic ischemic events [13]. Recent clinical studies have strengthened this concept by showing that non-stenotic carotid plaques may harbor high-risk morphological features detectable on advanced imaging, such as ulceration or intraplaque hemorrhage, which can increase embolic potential through mechanisms including plaque rupture, microthrombus formation, and artery-to-artery embolism despite <50% stenosis.

Herein, we evaluated the relationship between non-stenotic plaques and ESUS using computed tomography (CT) angiography (CTA). We predicted that in patients with ESUS of the anterior circulatory system, more non-stenotic plaques would exist in the carotid artery ipsilateral to the infarct than on the contralateral side.

## 2. Materials and Methods

We conducted a retrospective review of a comprehensive, single-center database that prospectively collected data on a consecutive series of patients who were hospitalized for acute stroke at our institution between April 2017 and December 2022; from this cohort, we identified and enrolled those who met the diagnostic criteria for ESUS during the study period according to predefined inclusion and exclusion criteria.

Inclusion criteria (entry) were

1. Age ≥ 18 years;
2. Diagnosis of unilateral anterior circulation ESUS according to the Cryptogenic Stroke/ESUS International Working Group criteria;
3. Underwent CTA during the index hospitalization.

Exclusion criteria (exit) were

4. ESUS involving posterior circulation;
5. Bilateral anterior circulation ESUS (excluded because our study aimed to evaluate the relationship between carotid small plaques and ESUS, and such plaques are unlikely to cause bilateral anterior circulation infarcts, making side-to-side comparison unfeasible);
6. Incomplete or poor-quality CTA images.

From an initial 71 patients with unilateral anterior circulation ESUS, 36 without CTA were excluded, leaving 35 patients for the final analysis. 

We diagnosed ESUS according to the Cryptogenic Stroke/ESUS International Working Group criteria as follows: (a) ischemic stroke detected using CT or magnetic resonance imaging (MRI) that is not lacunar; (b) absence of extracranial or intracranial atherosclerosis causing ≥ 50% luminal stenosis in the arteries supplying the area of ischemia; (c) no major risk of cardioembolic embolism source; and (d) stroke was not attributed to any other defined etiology, such as arteritis, arterial dissection, migraine with vasospasm, or substance abuse.

The degree of arterial stenosis was assessed using the North American Symptomatic Carotid Endarterectomy Trial (NASCET) criteria, which is a widely recognized and extensively utilized method for evaluating the severity of vascular narrowing [14]. The NASCET approach has been adopted in numerous clinical trials and research studies due to its standardized measurement technique and its ability to provide reproducible and clinically meaningful assessments of carotid artery stenosis.

CTA acquisition parameters were as follows: 64-slice multidetector CT scanner (Aquilion Precision; Canon Medical Systems), slice thickness of 0.625 mm, and intravenous administration of 70 mL of nonionic contrast medium (iopamidol, 370 mgI/mL) at a rate of 26–30 mgI/kg/sec, with imaging from the aortic arch to the circle of Willis using a bolus-tracking technique. CTA images were evaluated using axial, sagittal, and coronal source images with a standardized window level setting (WL: 300 HU, WW: 700 HU) optimized for vascular assessment.

CTA was performed within 7 days after symptom onset. We evaluated the presence or absence of non-stenotic carotid artery plaque lesions ipsilateral and contralateral to the cerebral infarction lesion using CTA and quantitatively compared the thickness of these plaques between the two sides.

All images were reviewed independently by two board-certified neurologists blinded to both the stroke side and all clinical data. Agreement between raters was evaluated with Cohen’s kappa coefficient (κ = 0.87), indicating strong agreement. In cases of disagreement, consensus was reached through joint review.

**Figure 1:**
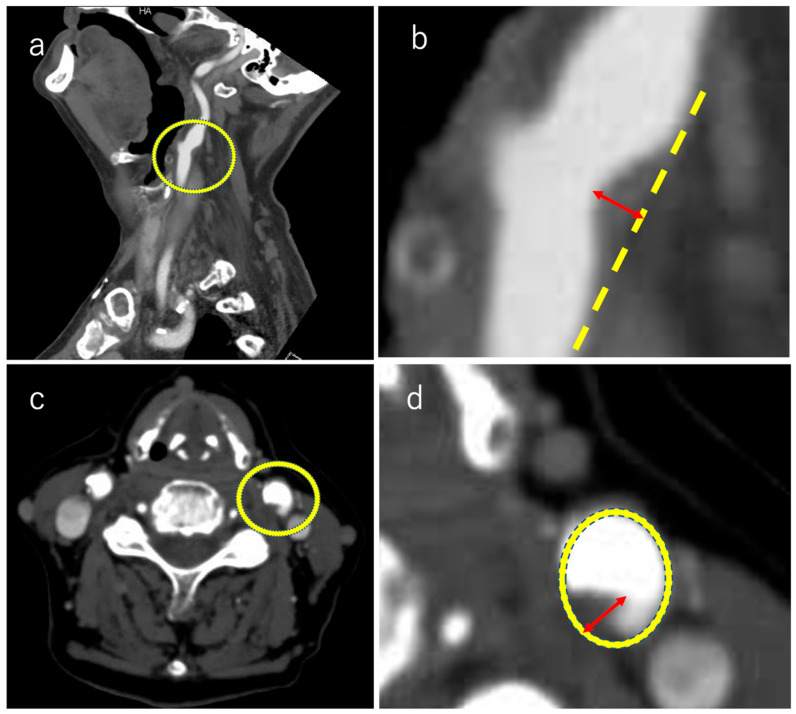
Measuring plaque size using computed tomography angiography. (**a**) Sagittal and (**b**) enlarged images of the plaque area. A line perpendicular to the vessel wall is drawn, and its maximum diameter is measured. Similarly, the axial view (**c**) and enlarged image of the plaque area is shown (**d**). A line perpendicular to the vessel wall is drawn to measure its maximum diameter.

Plaque thickness was measured manually using electronic calipers perpendicular to the vessel wall at the point of maximal protrusion into the lumen (Figure 1). To ensure consistency, we used a multiplanar reformation approach to select the axial slice that demonstrated the greatest plaque prominence. The largest part was considered as the maximum plaque diameter. Carotid plaque was considered present when a localized lesion projected into the arterial lumen and measured more than 1.2 mm in thickness [15]. For the primary analysis, we focused on plaques with a maximal thickness > 3 mm. This cutoff was selected based on prior imaging studies using MRI and carotid ultrasound, which showed that plaques ≥ 2.2–3.5 mm are more likely to harbor high-risk features such as intraplaque hemorrhage or a lipid-rich necrotic core [16,17]. We chose 3 mm as a conservative threshold that has also been applied in previous CTA-based studies [18], ensuring methodological consistency with the existing literature.

Additionally, we investigated whether additional non-stenotic plaques were present on the ESUS side than on the contralateral side. The side identified as the ESUS side corresponded to the hemisphere in which the cerebral infarction occurred, as confirmed by imaging findings.

Based on our study hypothesis, we anticipated that non-stenotic plaques would be more frequently observed on the ipsilateral side of the cerebral infarction, corresponding to the ESUS side. Additionally, we collected data regarding the medical history and baseline characteristics of each patient, including age, sex, National Institutes of Health Stroke Scale (NIHSS) score at admission, hypertension, diabetes, dyslipidemia, chronic kidney disease, smoking status, and history of alcohol consumption.

**Figure 2:**
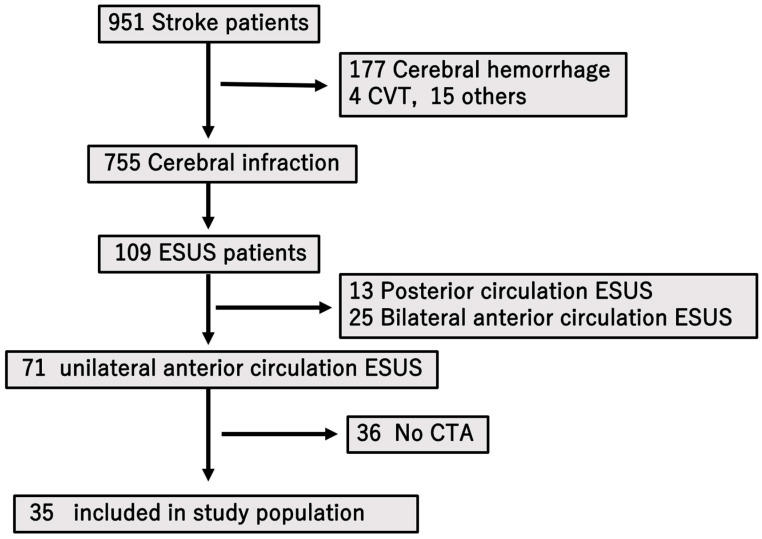
Flow chart illustrating the inclusion and exclusion criteria of the patients.

For clarity, Figure 1 and Figure 2 illustrate the plaque measurement methodology and the study flow, respectively.

### Statistical Analysis

We conducted a statistical analysis to evaluate differences in the presence of carotid artery plaques between the ESUS side and the contralateral (non-ESUS) side. Categorical variables were summarized and reported as absolute numbers accompanied by their corresponding percentages, whereas continuous data were described as mean ± standard deviation. Categorical comparisons were made using either the chi-squared test or Fisher’s exact test, selected according to the distribution of expected frequencies. For continuous variables, unpaired (independent samples) *t*-tests were applied to assess statistical differences between the two groups. All statistical analyses were conducted in a two-tailed manner, and results with a *p*-value below 0.05 were regarded as statistically significant.

## 3. Results

During the study period from April 2017 to December 2022, a total of 951 individuals were hospitalized at our center with a diagnosis of stroke. Among these, 755 patients were determined to have experienced cerebral infarction based on neuroimaging and clinical evaluation. Of this subgroup, 109 individuals met the diagnostic criteria for ESUS. After applying exclusion criteria—specifically eliminating patients with ESUS affecting the posterior circulation or those with bilateral anterior circulation involvement—a total of 71 patients were identified as having ESUS localized to the unilateral anterior circulation.

Subsequently, patients who had not undergone computed tomography angiography (CTA) were further excluded from the study population (*n* = 36). Ultimately, a final cohort of 35 patients who satisfied all inclusion criteria and underwent appropriate vascular imaging were enrolled for detailed analysis (Figure 2).

A total of 951 patients with stroke were hospitalized during the study period, of whom 755 had cerebral infarction. Among these, 109 were diagnosed with ESUS. After excluding cases involving posterior or bilateral anterior circulation, 71 patients with unilateral anterior circulation ESUS remained. Of these, 36 who did not undergo CT were excluded, leaving 35 patients enrolled in the study.

Within this cohort of 35 patients, we investigated the prevalence of carotid artery plaques exceeding 3 mm in maximum thickness, comparing findings between the side corresponding to the ESUS-related infarction and the contralateral, unaffected side. The proportion of patients with plaques greater than 3 mm was significantly higher on the ESUS side compared to the contralateral side, with prevalence rates of 31% (11 out of 35 patients) versus 8% (3 out of 35 patients), respectively. Statistical analysis revealed this difference to be significant (*p* = 0.01), with an odds ratio of 4.96 (95% confidence interval, 1.24–19.77), suggesting a notable lateral asymmetry in plaque distribution potentially associated with the infarct (Table 1).

In terms of quantitative plaque burden, the mean thickness of carotid artery plaques on the ESUS-affected side was calculated to be 1.93 ± 2.01 mm (mean ± standard deviation), with a median plaque thickness of 2.0 mm and an interquartile range (IQR) spanning from 0.0 to 3.3 mm. These findings indicate considerable variability in plaque size, including the presence of patients with notably large plaques. In contrast, the contralateral carotid artery demonstrated a reduced average plaque thickness of 1.09 ± 1.46 mm, accompanied by a median value of 0 mm and an IQR ranging from 0.0 to 2.8 mm. This distribution suggests that a significant portion of patients exhibited either minimal plaque accumulation or an absence of detectable plaques on the contralateral side. Although the side-by-side comparison in plaque thickness did not reach the threshold for statistical significance (*p* = 0.052), the observed directional trend supports the hypothesis that non-stenotic carotid plaques may more frequently localize to the side of the infarction.

To further explore the clinical relevance of carotid plaque burden, we compared baseline characteristics between two patient subgroups—those with and those without plaques measuring ≥ 3 mm on the ESUS side. Variables assessed included age, sex, NIHSS score at admission, and typical vascular risk factors including hypertension, dyslipidemia, diabetes mellitus, chronic kidney disease, smoking history, and alcohol use. Nevertheless, none of these variables showed statistically significant differences between the two groups (Table 2), indicating that carotid plaques ≥ 3 mm were not distinctly linked to recognizable baseline risk factor profiles within this cohort.

## 4. Discussion

In the present study, we demonstrated that patients diagnosed with embolic stroke of undetermined source (ESUS) were significantly more likely to exhibit non-stenotic carotid artery plaques measuring 3 mm or greater on the infarcted side compared to the contralateral side. This asymmetrical distribution of plaque burden provides new evidence supporting the hypothesis that non-stenotic carotid plaques—although traditionally regarded as having limited clinical significance compared to hemodynamically significant stenotic lesions—may play a more prominent and active role in the pathogenesis of ESUS than previously appreciated.

This finding carries important clinical implications, particularly given the fact that ESUS continues to pose a substantial diagnostic and therapeutic challenge in everyday stroke management. The term ESUS encompasses a heterogeneous group of patients, and the embolic sources in many of these cases often remain elusive, even after comprehensive diagnostic workup. In this context, identifying a plausible arterial embolic source—such as non-stenotic atherosclerotic plaque formation—could significantly enhance our mechanistic understanding of stroke etiology in ESUS and serve as a basis for improved clinical decision-making.

More specifically, recognizing these non-stenotic plaques as potential contributors to cerebral embolism may allow clinicians to more accurately stratify stroke risk in patients who lack conventional high-risk embolic sources, such as paroxysmal atrial fibrillation or severe carotid artery stenosis. Such insights could help refine current diagnostic frameworks and enable the development of more individualized secondary prevention strategies, tailored to the underlying mechanism of stroke in each patient.

The implication of our findings is that a substantial subset of patients who are currently grouped under the broad and often ambiguous category of ESUS may actually harbor covert atherosclerotic mechanisms that are not detected by conventional criteria focused primarily on the degree of stenosis. These subclinical atherogenic sources could represent actionable targets for therapy, particularly through intensified antiplatelet regimens or more aggressive lipid-lowering strategies aimed at plaque stabilization. This evolving paradigm—shifting from a purely stenosis-based approach to one that incorporates detailed plaque morphology and vulnerability—has the potential to significantly improve clinical outcomes by enabling more precise, mechanism-specific, and individualized treatment approaches for this complex and heterogeneous patient population.

According to widely accepted classification criteria for ischemic stroke, such as the TOAST (Trial of Org 10172 in Acute Stroke Treatment) criteria, carotid atherosclerotic disease is generally considered a causative embolic source only when the degree of stenosis reaches 50% or greater [10]. This threshold has been established based on evidence linking significant luminal narrowing to an increased risk of cerebral infarction due to artery-to-artery embolism or hemodynamic compromise. Even within the diagnostic framework for ESUS, stenosis less than 50% in the proximal vessels, including the common and internal carotid arteries, is typically not regarded as a potential embolic source [11]. This conventional cutoff, however, may overlook the potential role of non-stenotic plaques, which do not meet the severity criteria for stenosis but may still contribute to cerebral embolism through mechanisms such as plaque rupture, ulceration, or thrombogenicity. Therefore, while the presence of ≥50% stenosis remains an important indicator in clinical practice for attributing stroke etiology to carotid atherosclerosis, there is growing recognition that plaques causing less than 50% stenosis might also be clinically relevant and warrant further investigation.

In recent years, this notion has gained increasing support from a number of observational and imaging studies, which suggest that the risk posed by non-stenotic carotid plaques may be considerably greater than previously considered. Supporting this evolving perspective, several recent studies have reported that even non-stenotic small plaques with a stenosis rate of less than 50% can serve as embolic sources of cerebral infarction [12].

Our findings are consistent with these previous clinical studies in demonstrating a higher prevalence of ipsilateral non-stenotic plaques in patients with ESUS. However, our work extends prior evidence by using CTA—a widely available, standardized, and reproducible modality—instead of ultrasound or MRI, thereby highlighting the feasibility of incorporating plaque assessment into routine acute stroke imaging. This practical advantage could facilitate broader application in clinical settings, particularly in centers without ready access to advanced plaque imaging techniques. Of these mechanisms, increasing focus has been directed toward the link between non-stenotic plaques and ESUS, since accumulating evidence indicates that such lesions may act as hidden arterial sources of embolism in this subtype of stroke. These findings challenge the long-held assumption that only hemodynamically significant stenosis is clinically relevant. For example, a high-resolution MRI-based plaque imaging study demonstrated that only carotid plaques with a thickness of ≥2.2 mm exhibited imaging features characteristic of unstable plaques, such as intraplaque hemorrhage or lipid-rich necrotic cores [19], highlighting that plaque size—and more importantly, plaque vulnerability—may serve as a more accurate indicator of embolic potential than stenosis severity alone.

Previous reports that examined the size threshold differentiating asymptomatic from symptomatic carotid plaques in patients with carotid artery stenosis have also reported cutoff values of ≥2.2 mm and ≥3.5 mm, further reinforcing the clinical significance of plaque thickness in risk stratification and suggesting that even moderately sized plaques may have pathogenic potential depending on their composition and biological behavior [16,20]. A study using cervical ultrasonography to examine small plaques in patients with ESUS suggested that plaques > 2.6 mm may be an embolization source [17]. These studies used MRI or cervical ultrasound to evaluate carotid artery plaques; however, reports using CT angiography to evaluate plaques in patients with ESUS like this study are limited. Only Jonathan M et al. reported that the carotid artery on the infarcted side was more likely to have plaques of ≥3 mm than those on the contralateral side in CT angiography [18].

CTA has several advantages in that it does not require any special examination techniques and demonstrates high inter-examiner reliability, unlike ultrasonography, which is more operator-dependent. Furthermore, many medical institutions already widely utilize CTA as a routine imaging modality. In addition, the relatively short examination time makes CTA particularly suitable for use in the acute phase of cerebral infarction, where rapid evaluation is essential. Compared to MRI and ultrasonography, CTA provides superior visualization and an easier evaluation of calcified vascular lesions, which can be challenging to assess accurately by those modalities. In addition, CTA allows for a comprehensive evaluation of vascular territories that are challenging to examine with ultrasound or MRA, including the proximal part of the common carotid artery and the distal internal carotid artery segment adjacent to the skull base. Taking all these advantages into consideration, we believe that CTA is a highly useful tool for evaluating potential embolic sources in patients diagnosed with ESUS.

Recently, the clinical concept of symptomatic non-stenotic carotid disease (SyNC) has been introduced to characterize patients who experience ischemic cerebrovascular events despite the lack of substantial narrowing in the carotid artery. This framework acknowledges that non-stenotic carotid plaques may nevertheless possess high-risk features, such as plaque instability or thrombogenic potential, which contribute to stroke pathogenesis [13,21]. This emerging paradigm aligns closely with our findings, emphasizing that comprehensive stroke risk assessment should incorporate not only the degree of luminal narrowing but also detailed plaque morphology and stability, thereby broadening the understanding of embolic mechanisms beyond traditional stenosis thresholds. Incorporating SyNC into the ESUS framework could bridge the gap between so-called cryptogenic and atherosclerotic strokes, prompting a reclassification of some ESUS cases and a shift in management strategies. Our data support this view, demonstrating a side-to-side difference in plaque thickness consistent with a possible causal role of non-stenotic lesions. Looking ahead, our findings suggest several possible clinical applications if confirmed by larger, prospective studies. First, the CTA-based detection of non-stenotic carotid plaques could be incorporated into the acute evaluation of ESUS patients, enabling earlier identification of those with potentially high-risk atherogenic mechanisms. Second, such imaging could support more precise risk stratification and help guide individualized secondary prevention strategies—such as intensified antiplatelet therapy or lipid-lowering interventions—targeted at patients with high plaque burden despite the absence of significant stenosis. Finally, standardized CTA plaque assessment protocols could be developed for routine stroke workup, making it feasible for a wide range of medical centers, including those without access to high-resolution MRI or specialized ultrasound techniques, to integrate plaque morphology into decision-making frameworks.

In the present study, we found that the presence or absence of carotid artery plaques measuring 3 mm or greater on the ESUS side was not associated with any significant differences in traditional vascular risk factors or baseline clinical characteristics, including hypertension, diabetes mellitus, dyslipidemia, chronic kidney disease, and lifestyle factors such as smoking and alcohol consumption. Previous studies have reported that males tend to have a higher prevalence of non-stenotic carotid artery plaques compared to females. Furthermore, these studies have indicated that there are notable sex differences in the risk factors associated with non-stenotic carotid plaques, where aging and hypertension are considered the primary risk factors in male patients, whereas tobacco use is recognized as a more prominent risk factor among female patients [22]. However, in our present study, we were unable to identify any clear or statistically significant risk factors associated with non-stenotic carotid artery plaques. This limitation primarily stems from the relatively small sample size, which unfortunately did not allow for subgroup analyses stratified by sex to investigate potential differences in risk factors. Consequently, although prior research highlights the importance of sex-specific risk factors in ESUS patients, our study lacked sufficient power to explore these differences. Future studies with larger cohorts are warranted to better elucidate sex-related variations in risk factors for non-stenotic carotid artery plaques.

Our findings should be interpreted considering several important limitations, which are summarized below. First, our small sample size (*n* = 35) substantially limits statistical power, particularly for subgroup analyses. The results should therefore be considered hypothesis-generating and interpreted with caution. Larger, prospective, multicenter studies are needed. Second, we assessed plaque size only without evaluating morphological or compositional characteristics that are critical for determining plaque vulnerability. Features such as intraplaque hemorrhage, ulceration, fibrous cap rupture, and a lipid-rich necrotic core are well-established predictors of embolic potential. However, standard CTA, as applied in this study, cannot reliably characterize these features, which require advanced high-resolution MRI or targeted carotid ultrasound techniques. This methodological limitation means that our results capture plaque burden but not plaque vulnerability, which is essential for mechanistic insight and clinical decision-making. Consequently, the absence of plaque vulnerability data represents one of the most significant limitations of this study. Although future prospective studies incorporating carotid ultrasound or MRI could address this issue, such data were not available for the present cohort, and adding them retrospectively would require a substantial protocol amendment and introduce potential selection bias. Third, although we compared classical vascular risk factors, we could not account for unmeasured confounders such as systemic inflammation, plaque calcification, or genetic predispositions, which may influence the results. Fourth, regarding imaging evaluation, carotid artery plaque size was measured in three anatomical planes—axial, sagittal, and coronal. However, this approach does not fully capture the complex three-dimensional morphology and spatial distribution of plaques. Although advanced three-dimensional imaging techniques would provide greater accuracy, they are technically demanding, time-consuming, and difficult to apply in routine practice. Therefore, we adopted a simplified yet practical method that offers a reasonable balance between accuracy and clinical feasibility. Fifth, MRI and carotid ultrasound can provide a detailed characterization of plaque vulnerability. MRI is superior in detecting intraplaque hemorrhage, while ultrasound allows for real-time hemodynamic assessment. CTA, however, is widely available, quick, and highly reproducible, making it practical in acute stroke evaluation despite its limited capacity for compositional analysis.

Finally, this was a single-center, retrospective study conducted in a Japanese cohort, excluding posterior circulation and bilateral ESUS cases. These factors limit external validity, and findings may not be generalizable to other populations or stroke subtypes.

To address these limitations, future studies employing prospective study designs are essential. In particular, large-scale, multicenter prospective research would provide more robust evidence by minimizing selection bias and increasing the generalizability of findings. Such well-designed prospective investigations would also allow for a more precise evaluation of carotid plaque characteristics using advanced imaging modalities, thereby contributing to a deeper understanding of their clinical significance and improving patient care.

In addition, our study highlights important research gaps in this field. First, there is a scarcity of studies evaluating symptomatic non-stenotic carotid disease (SyNC) using CTA, particularly in the context of ESUS. Second, most existing data are derived from single-center, retrospective analyses, which limit the generalizability of findings. Third, there is a need for future multicenter, prospective studies incorporating advanced imaging modalities—such as high-resolution MRI or targeted carotid ultrasound—to better characterize plaque morphology and vulnerability. By identifying these gaps, our study helps set a research agenda for future investigations.

Our study addresses one of these gaps by providing preliminary evidence that non-stenotic carotid plaque thickness, as assessed by CTA, is associated with the side of infarction in ESUS. This relationship has been underexplored in the literature, and our findings contribute to a growing body of evidence supporting the role of small, non-stenotic plaques as potential embolic sources. These results may inform future research aimed at refining diagnostic criteria for ESUS and guiding mechanism-specific preventive strategies.

## 5. Conclusions

Patients with ESUS were more likely to demonstrate non-stenotic plaques of ≥3 mm within the carotid artery on the infarcted side compared with the carotid artery on the opposite side. These findings support the concept that small, non-stenotic plaques may be a source of embolism. However, given the limited sample size, a lack of plaque vulnerability assessment, and restricted generalizability, our results should be interpreted cautiously and viewed as hypothesis-generating. Larger, prospective, multicenter studies with comprehensive plaque characterization are warranted.

## Figures and Tables

**Table 1:** Prevalence of plaques > 3 mm in the embolic stroke of undetermined source and contralateral sides of the carotid arteries.

	Embolic Stroke of Undetermined Source Side	Contralateral Side	OR (95%CI)	*p*-Value
Plaque ≥ 3 mm	11 (31%)	3 (8%)	4.96 (1.24–19.77)	0.01

**Table 2:** Background of the patients with plaques > 3 mm.

	Plaque (+) (*n* = 11)	Plaque (−) (*n* = 24)	*p*-Value
Male, *n* (%)	8 (72)	11 (45)	0.13
Age (mean ± SD) years	72.9 ± 17	65.0 ± 14.7	0.20
NIHSS (mean ± SD) points	4.1 ± 2.8	5.3 ± 7.8	0.51
Hypertension, *n* (%)	10 (92)	19 (79)	0.41
Dyslipidemia, *n* (%)	5 (45)	12 (50)	0.90
Diabetes, *n* (%)	4 (36)	6 (25)	0.77
CKD, *n* (%)	2 (18)	2 (8)	0.57
Smoker, *n* (%)	3 (27)	12 (50)	0.28
Alcoholic, *n* (%)	3 (27)	4 (16)	0.65

NIHSS, National Institutes of Health Stroke Scale; AF, atrial fibrillation; CKD, chronic kidney disease.

## Data Availability

The data that support the findings of this study are not publicly available because they contain information that could compromise the privacy of research participants but are available from the corresponding author upon reasonable request.
